# Utility of the combination of IVIM-DWI MRI and baseline eGFR for identifying a high risk of chronic kidney disease progression

**DOI:** 10.3389/fmed.2025.1532210

**Published:** 2025-02-19

**Authors:** Yazhen Yu, Wei Zhang, Lina Zhu, Han Zhou, Shaoshan Liang, Longjiang Zhang, Zhihong Liu, Jiong Zhang

**Affiliations:** ^1^National Clinical Research Center of Kidney Disease, Jinling Hospital, The First School of Clinical Medicine, Southern Medical University, Nanjing, China; ^2^Department of Radiology, Jinling Hospital, Nanjing Medical University, Nanjing, China; ^3^National Clinical Research Center of Kidney Disease, The Affiliated Jinling Hospital of Nanjing Medical University, Nanjing, China

**Keywords:** chronic kidney disease, pathology, intravoxel incoherent motion, diffusion-weighted imaging, prognosis

## Abstract

**Background:**

Currently, the baseline estimated glomerular filtration rate (eGFR), the urine albumin level and renal fibrosis are the common risk and prognostic factors for chronic kidney disease (CKD). Intravoxel incoherent motion (IVIM) diffusion-weighted imaging (DWI) is a proven noninvasive tool for assessing renal fibrosis. The aim of this study was to evaluate whether IVIM-DWI could be used to identify high-risk patients with CKD during long-term follow-up.

**Methods:**

In this exploratory study, 62 CKD patients who were followed for 5 years and who underwent renal biopsy and IVIM-DWI magnetic resonance imaging (MRI) at the National Clinical Research Center of Kidney Disease in China were enrolled. We recorded baseline data, including clinical, pathology and MRI parameters, and evaluated the associations between baseline parameters and renal outcomes. The value of DWI parameters in predicting end-stage kidney disease (ESKD) was compared with that of clinical and pathological data.

**Results:**

The mean baseline eGFR was 78.1 ± 28.05 ml/min/1.73 m^2^, and the median eGFR slope was −0.07 (−0.43–0.06) ml/min/1.73 m^2^/yr. Sixteen patients eventually developed ESKD. The values of perfusion fraction (f) were positively correlated with the eGFR slope (*r_s_* = 0.54, *p* = 0.028). The results of the receiver operating characteristic (ROC) analysis demonstrated that the areas under the curve (AUCs) of total apparent diffusion coefficient (ADC_T_), true diffusion coefficient (D) and f in distinguishing ESKD were 0.778 (95% confidence interval [95% CI] 0.65–0.906; *p* = 0.001), 0.893 (95% CI 0.816–0.97; *p* <0.001), and 0.823 (95% CI 0.706–0.939; *p* < 0.001), respectively. For the combination of baseline eGFR with both D and f, the AUC was significantly greater than that for the combination of baseline eGFR and interstitial extracellular matrix volume [AUC 0.955 (95% CI, 0.909 to 1.000) vs. AUC 0.886 (95% CI, 0.803 to 0.969), *p* = 0.049]. Cox proportional hazard regression revealed that f was a risk and prognostic factor for ESKD after adjustment for baseline variables (*p* = 0.006).

**Conclusion:**

The combination of baseline eGFR and IVIM-DWI outperforms pathological factors alone in the diagnosis of long-term kidney dysfunction. This study indicated that IVIM-DWI could be a promising tool for identifying patients at high risk of CKD progression.

## Introduction

Chronic kidney disease (CKD) is a progressive condition that causes not only kidney failure resulting in dialysis or transplantation but also cardiovascular disease, infections and death (1). The assessment of interstitial fibrosis with renal biopsy plays an important role in the identification of kidney function decline in CKD patients. However, owing to the invasiveness and complications of kidney biopsy, some patients may refuse surgery, and their kidney prognosis remains unclear. Therefore, there is a need to identify noninvasive biomarkers that could serve as risk factors for the progression of CKD to allow clinicians to manage patients properly. The estimated glomerular filtration rate (eGFR) and urine albumin level are readily available clinical parameters that are currently used to identify the risk of chronic kidney disease progression (2–4).

Diffusion-weighted magnetic resonance imaging (DWI-MRI) is a highly sensitive method for detecting the movement of water molecules in tissue and can indirectly indicate renal damage (5). Evidence from other studies has shown that DWI can be used to reflect renal function and pathology in CKD patients (6). Intravoxel incoherent motion DWI (IVIM-DWI) is a technique developed in recent years that can quantify microperfusion and diffusion separately with a biexponential model (7). Our study was designed on the basis that that IVIM-DWI can be used to effectively evaluate fibrosis in the kidney, as shown by the graphs of the mapping model from imaging and the eGFR into fibrosis (8, 9). Additionally, previous studies have shown that IVIM-DWI parameters can be used to assess fibrosis and evaluate kidney function decline in native and transplanted kidneys (9–11). However, whether functional MRI biomarkers can predict the risk of kidney function decline in long-term follow-up for CKD patients remains unclear, and cross-sectional comparisons with pathological findings are lacking. Therefore, this study aimed to investigate the value of IVIM-DWI in the prognosis of CKD patients.

## Materials and methods

### Study population

We collected data from a group of adult CKD patients who underwent IVIM-DWI and kidney biopsy from January 2016 to December 2017 and visited the National Clinical Research Center of Kidney Disease, Jinling Hospital, for 5 years. Among the 97 participants with available baseline data, 62 were included in the study after 35 were excluded because of data integrity and availability concerns (Supplementary Figure 1). MRI was scheduled either 1 day before or within 1 week after the biopsy. The main exclusion criteria included refusal to participate in the study, claustrophobia or other standard MRI contraindications, and acute kidney injury confirmed by biopsy. This research was approved by the local ethics committee, and all included individuals provided informed consent to participate in our study (2024DZKY-053-01).

### Clinical and laboratory assessments, follow-up, and outcomes

Clinical and laboratory data were collected at the time of MRI scanning. The data we recorded were as follows: age, sex, serum creatinine (Scr) level and 24-h urine protein. The eGFR was calculated via the CKD Epidemiology Collaboration equation (CKD-EPI), namely, eGFR (ml/min/1.73 m^2^) =141 × min (Scr/*κ*)*^α^* × max(Scr/κ)^-1.209^ × 0.993^Age^ [×1.018 if female], where age is in years, Scr is in mg/dl. (*κ* = 0.7 for females and 0.9 for males; α = −0.329 for females and − 0.411 for males; min indicates the minimum of Scr/κ or 1, and max indicates the maximum of Scr/κ or 1). Scr was measured at baseline and at follow-up visits every 6 months for the first year and every 12 months until the end of the study. The eGFR slope was calculated by using regression analysis using all available data during each visit, which can account for the variability in the eGFR. The renal outcome included the rate of eGFR change over 5 years and end-stage kidney disease (ESKD), which was defined as an eGFR decline >30% or the need for regular renal replacement therapy. For those who progressed to ESKD, the last available data were recorded. If no eGFR value was available, we used a value of 10 mL/min/1.73 m^2^ to assess the primary outcome. During the study, at least three eGFR values, including baseline values, were obtained for each participant.

### Histological fibrosis quantification

Pathologic diagnoses and preliminary lesion identifications were provided by an experienced nephropathologist who was blinded to the functional MRI results. Kidney fibrosis was quantified via Masson’s trichrome-stained kidney sections as previously reported (12). The sections were scanned on a digital pathology platform (Aperio Scanscope XT Turbo Scanner; Leica, Wetzlar, Germany) and analyzed with ImageScope (Aperio). Kidney fibrosis was then assessed quantitatively by the percentage area that appeared brilliant green (9, 12).

### MRI acquisition and analysis

**Figure 1:**
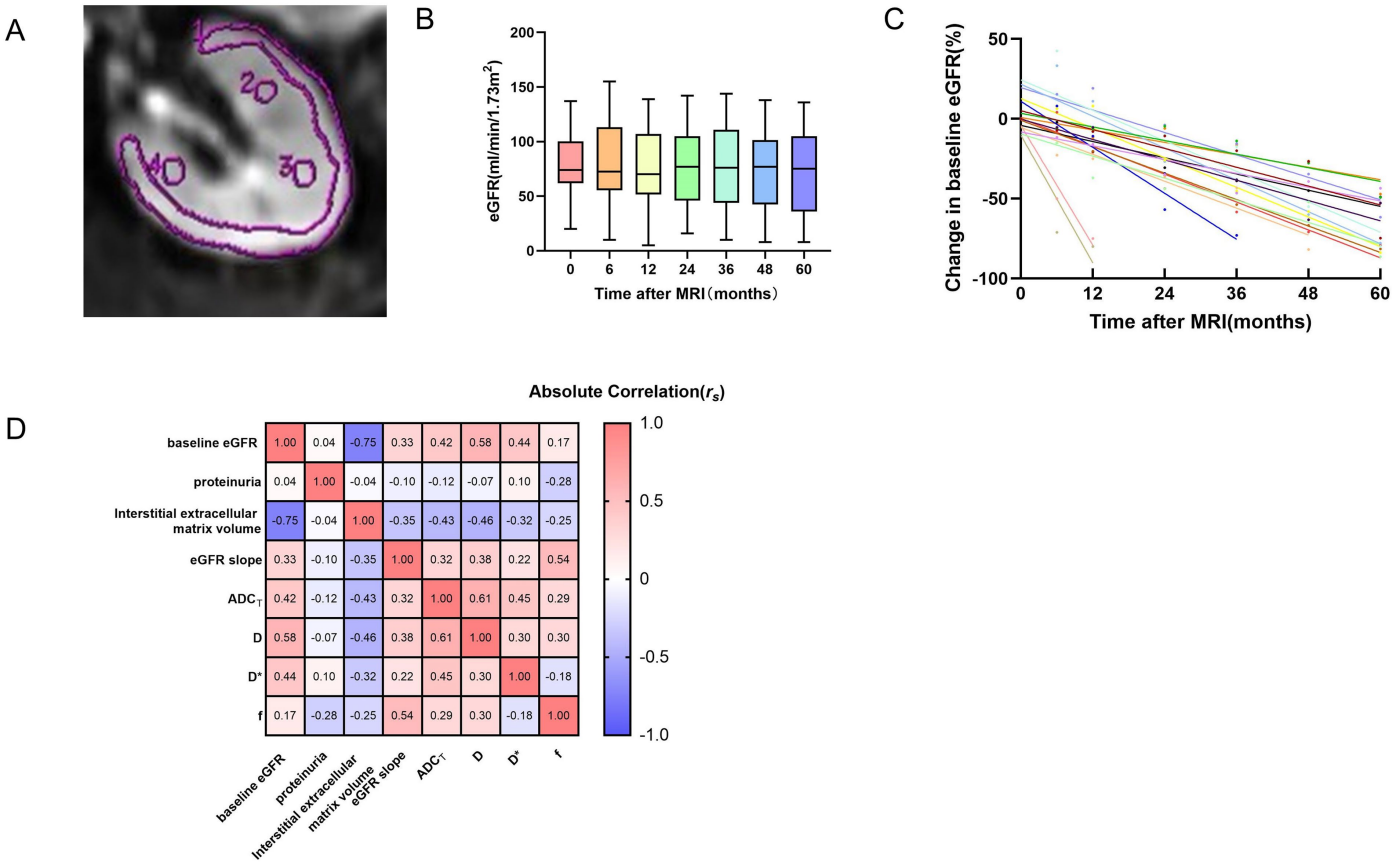
ROI placement for kidney IVIM-DWI **(A)**, time courses of the eGFR for all patients over the 5 years **(B)**, time curves of patients with an eGFR decline >30% **(C)**, correlation heatmap of multiparametric MRI and clinicopathological variables **(D)**. **(A)** The single ROI should be as large as possible in the cortex. Three elliptical ROIs were placed on each layer, and the average was taken. **(B)** The box plot shows that the change in the eGFR for patients over the 5 years was relatively flat. The number of patients at each time point was 62, 62, 62, 59, 58, 57, and 55, respectively. The different colors represent the eGFR values of all patients at different time points. **(C)** Shown are eGFR trajectories over the follow-up period, normalized to baseline eGFR, for patients with an eGFR decline >30% (n = 17). The different colors of data points and best fit lines represent the eGFR change in different patients. **(D)** The values within the rectangular boxes are the *r_s_* values for the Spearman correlation coefficient. eGFR, estimated glomerular filtration rate; ADC_T_, total apparent diffusion coefficient; D, true diffusion coefficient; D*, pseudo-diffusion coefficient; f, perfusion fraction.

All the scans were performed with a 3.0 Tesla MRI system (Discovery MR 750; GE Medical Systems, Milwaukee, WI, USA) equipped with a 32-channel body coil after the participants fasted for 4–6 h. Prior to the scans, the participants underwent training on how to hold their breath as instructed by the doctor during the examination. The relevant MRI parameters are summarized in Supplementary Table 1. The original MRI data were transmitted to a workstation (Functool 9.4.05, Advantage Workstation Volume Share 5, GE Healthcare), where the images were analyzed by two of the authors, who were blinded to the other results. Diffusion values were calculated on a pixel-by-pixel basis in 2 ways, as previously reported by our center (13). Regions of interest (ROIs) were placed to cover the corresponding renal cortex to the greatest extent possible at three levels while avoiding cystic areas and artifacts produced by breathing (Figure 1A). The total apparent diffusion coefficient (ADC_T_), true diffusion coefficient (D), pseudo-diffusion coefficient (D*) and perfusion fraction (f) were measured in the ROIs. The final values reflecting whole-kidney diffusion were obtained from the average values from the three layers of the cortex of the left kidney, as it was from that organ that the biopsy samples were obtained.

### Statistical analysis

The statistical analyses were performed using SPSS software (version 26.0; IBM) and MedCalc software (version 23.0.6). The Shapiro–Wilk normality test was performed to determine the normality of the data distribution. Continuous variables are presented as the means ± standard deviations for normally distributed data or medians and interquartile ranges for nonnormally distributed data. Spearman correlation analysis was used to assess the pairwise relationships among DWI parameters, clinical parameters and the interstitial extracellular matrix volume. *Post hoc* multiple pairwise comparisons were performed using the Bonferroni correction. The performance of the different variables in predicting ESKD was evaluated via receiver operating characteristic (ROC) curve analysis. Comparisons of areas under the ROC curve (AUCs) were performed via the Delong test. Cox regression was performed to analyze the risk factors for ESKD. The results were considered significant when the two-sided *p* value was <0.05.

## Results

### Study population

The baseline demographic and clinicopathological data of the study population obtained with functional MRI are summarized in Table 1. Sixty-two participants, including 35 males and 27 females with a median age of 39.5 (26.75–49) years, were included because they met our criterion and had complete data. The mean baseline eGFR was 78.1 ± 28.05 ml/min/1.73 m^2^, and the median eGFR slope was −0.07 (−0.43–0.06) ml/min/1.73 m^2^/yr. The time courses of the eGFRs for all patients over the 5 years are presented in Figures 1B,C. The causes of CKD included primary glomerular disease, secondary kidney disease, metabolically related kidney damage and renal tubulointerstitial disease (Supplementary Figure 2). Unfortunately, 16 patients progressed to ESKD and required regular dialysis during the follow-up period.

**Table 1:** Baseline demographic and clinicopathological data of all participants.

	All participants (*n* = 62)
Male/female	35/27
Age, year	39.5(26.75–49)
Serum creatinine, mg/dl	1.16(0.85–1.43)
Baseline eGFR, ml/min/1.73 m^2^	78.1 ± 28.05
Proteinuria, g/24 h	1.73(0.99–4.6)
ADC_T_, ×10^−3^ mm^2^/s	1.98 ± 0.24
D, ×10^−3^ mm^2^/s	1.43(1.34–1.51)
D*, ×10^−3^ mm^2^/s	12.47(9.3–25.21)
f	0.36(0.32–0.41)
Interstitial extracellular matrix volume, %	16.98(8.75–31.54)

eGFR, estimated glomerular filtration rate; ADC_T_, total apparent diffusion coefficient; D, true diffusion coefficient; D*, pseudo-diffusion coefficient; f, perfusion fraction. The distributions of baseline eGFR and ADCT revealed no remarkable skewness, so the data are presented as the means ± standard deviations, whereas the data for age, serum creatinine, proteinuria, D, D*, f and interstitial extracellular matrix volume were expressed as medians and interquartile ranges for nonnormally distributed data.

### Correlation analysis

The relationships between the DWI metrics and clinicopathological parameters are presented in Figure 1D and Supplementary Table 2. The baseline eGFR was positively correlated with the ADC_T_ (*r_s_* = 0.42, *p* = 0.028), D (*r_s_* = 0.58, *p* = 0.028) and D* (*r_s_* = 0.44, *p* = 0.028). As the eGFR slope decreased, f (*r_s_* = 0.54, *p* = 0.028) also decreased. In addition, the interstitial extracellular matrix volume was negatively correlated with the ADC_T_ (*r_s_* = −0.43, *p* = 0.028) and D (*r_s_* = −0.46, *p* = 0.028). The level of urine protein was not significantly correlated with DWI metrics.

### IVIM-DWI diagnostic parameters in early fibrosis

The Oxford classification of IgA nephropathy calculates interstitial fibrosis as interstitial fibrosis (0%), mild (1–25%), moderate (26–50%), or severe (≥51%) in the kidney (14). The criteria also apply to other chronic kidney diseases. For the purpose that early detection of fibrosis can help early intervention and delay the occurrence of ESKD, we analyzed the ability of DWI parameters to diagnose early fibrosis. As shown in Supplementary Table 3, the ADC_T_ and D had good performance in predicting ≤25% versus >25% interstitial extracellular matrix volume, with AUCs of 0.715 (*p* = 0.005) and 0.72 (*p* = 0.004), respectively. The ADC_T_ had a higher sensitivity, 87.5%, but a lower specificity, 54.5%, although there was no significant difference between the ADC_T_ and D in terms of the AUC (*p* > 0.05). Nevertheless, D* and f could not significantly predict early fibrosis in CKD patients.

### Diagnostic models of ESKD

**Figure 2:**
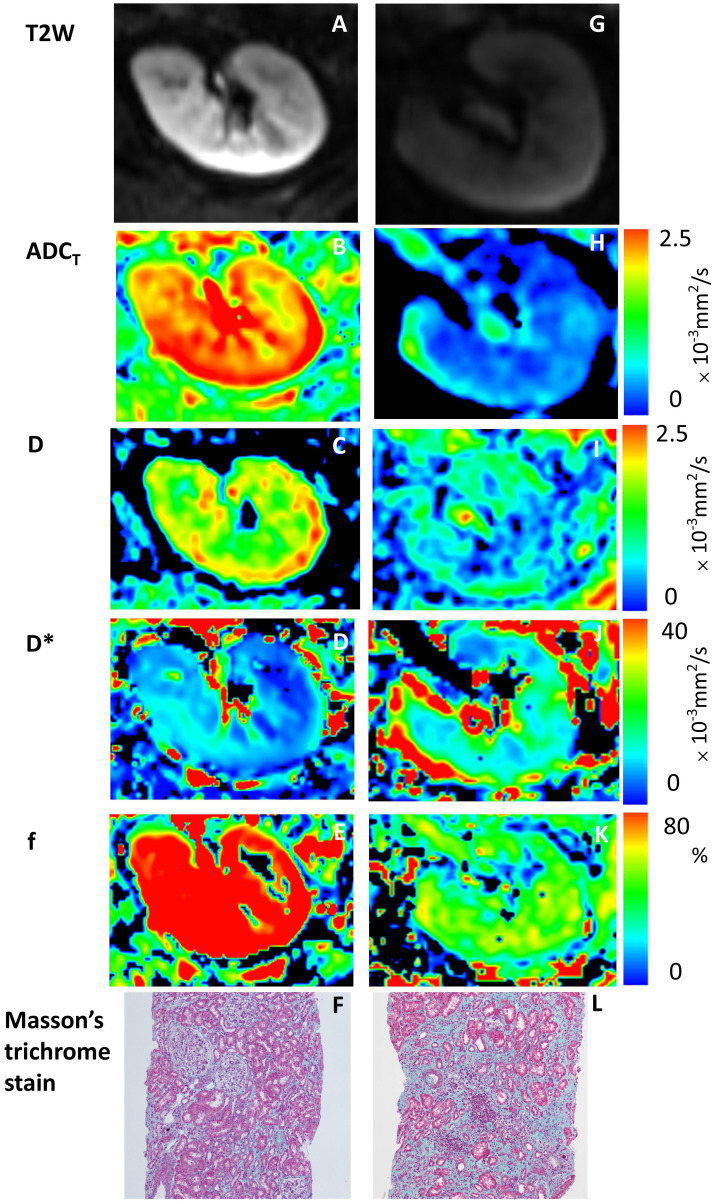
IVIM-DWI and pathological images of 2 typical patients with different prognoses. Panels **(A–F)** are from a 34-year-old man with stable kidney function, whereas panels **(G–L)** are from a 41-year-old man with ESKD as a renal outcome. **(A,G)** T2W maps; **(B,H)** ADC_T_ maps; **(C,I)** D maps; **(D,J)** D* maps; **(E,K)** f maps; **(F,L)** Masson trichrome stain (original magnification, ×100). Higher IVIM-DWI parameter values are shown with lower interstitial extracellular matrix volumes and stable kidney function, and high signal areas (red areas) are observed on the maps (the color bar on the right shows corresponding values of ADC_T_, D, D* and f).

**Figure 3:**
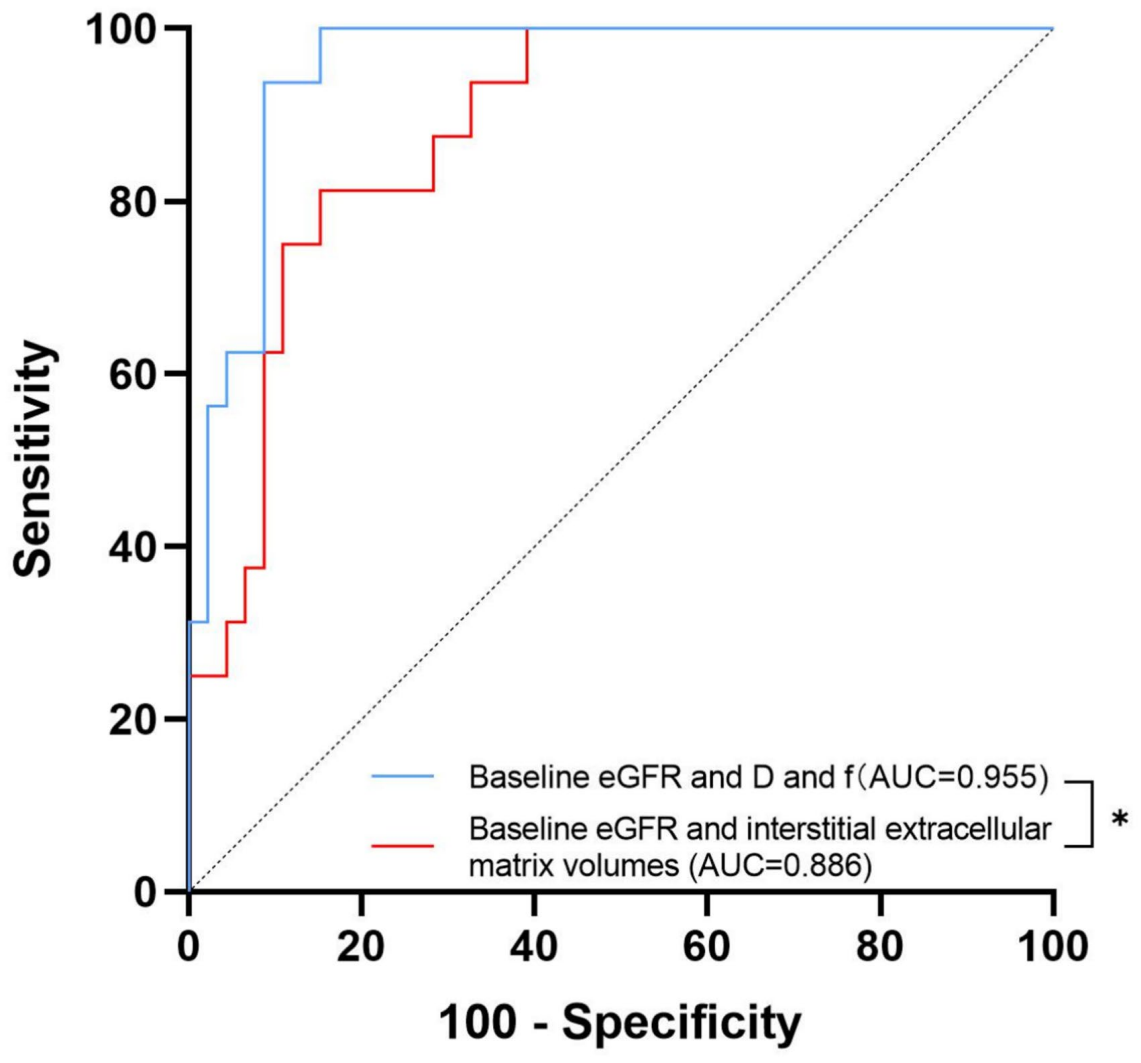
Receiver operating characteristic curves of the combined diagnosis of different measures. The area under the receiver operating characteristic curve (AUC) values of the combination of the baseline eGFR with both D and f and the combination of the baseline eGFR and interstitial extracellular matrix volume to distinguish ESKD were 0.955 and 0.886, respectively. * denotes *p* < 0.05.

A visual comparison of the IVIM-DWI and pathological images of 2 typical patients with different prognoses is shown in Figure 2. Clinical, pathological and imaging parameters were assessed to compare their diagnostic performance in predicting ESKD. The results of the ROC analysis are presented in Table 2 and Figure 3. Among clinicopathological parameters, the baseline eGFR and interstitial extracellular matrix volume had excellent discrimination ability for diagnosing ESKD, with AUCs of 0.885 (95% confidence interval [95% CI] 802–0.967, *p* < 0.001) and 0.833 (95% CI 0.727–0.939, *p* <0.001), respectively. For the DWI metrics, the AUCs for ADC_T_, D and f in distinguishing ESKD were 0.778 (95% CI 0.65–0.906; *p* = 0.001), 0.893 (95% CI 0.816–0.97; *p* <0.001), and 0.823 (95% CI 0.706–0.939; *p* <0.001), respectively. Compared with the interstitial extracellular matrix volume, D had a larger AUC and a higher sensitivity of 93.8% but a lower specificity of 78.3%. Although D had the largest AUC among these measures, the differences in the AUCs of the other measures were not significant (all *p* > 0.05). In the subsequent analysis, parameters with an AUC greater than 0.8 were selected to assess their joint diagnostic capability. Following clinical experience, the baseline eGFR and interstitial extracellular matrix volume were combined and demonstrated a large AUC of 0.886 (95% CI, 0.803 to 0.969; *p* < 0.001). For the combination of the baseline eGFR with both D and f, the AUC was significantly greater than that for the combination of the baseline eGFR and the interstitial extracellular matrix volume [AUC 0.955 (95% CI, 0.909 to 1.000) vs. AUC 0.886 (95% CI, 0.803 to 0.969), *p* = 0.049].

**Table 2:** ROC analysis of different measures to predict ESKD.

Measures	Cutoff	AUC (95% CI)	Sensitivity	Specificity	*p*
Separate diagnosis					
Baseline eGFR	69.5	0.885 (0.802 to 0.967)	87.5%	80.4%	<0.001
Urine protein	/	/	/	/	0.359
Interstitial extracellular matrix volume	31.58	0.833 (0.727 to 0.939)	62.5%	89.1%	<0.001
ADC_T_	1.84	0.778 (0.65 to 0.906)	62.5%	87%	0.001
D	1.39	0.893 (0.816 to 0.97)	93.8%	78.3%	<0.001
D*	/	/	/	/	0.094
f	0.33	0.823 (0.706 to 0.939)	68.8%	87%	<0.001
Combined diagnosis					
Baseline eGFR and interstitial extracellular matrix volume	/	0.886 (0.803 to 0.969)	81.3%	84.8%	<0.001
Baseline eGFR and D and f	/	0.955 (0.909 to 1.0)	93.8%	91.3%	<0.001

AUC, area under the curve; 95% CI, 95% confidence interval. The cutoff values of ADC_T_, D, and D* are presented as ×10^−3^ mm^2^/s; the baseline eGFRs are presented in ml/min/1.73 m^2^, and f is presented as a percentage.

### Prognostic value of IVIM-DWI

Cox regression analysis was performed to determine which functional MRI factors were associated with ESKD. The results are summarized in Table 3. According to the univariate analyses, ADC_T_, D and f were found to be associated with CKD prognosis. After adjustment for age, sex and baseline eGFR, ADC_T_ [hazard ratio (HR) 0.961 (95% CI, 0.926 to 0.998), *p* = 0.039], D [HR 0.95 (95% CI, 0.903 to 1.000), *p* = 0.049] and f [HR 0.835 (95% CI, 0.743 to 0.938), *p* = 0.002] were still significantly associated with ESKD. However, after further adjustment for the degree of albuminuria, the value of ADC_T_ and D in predicting ESKD disappeared (*p* = 0.072 and *p* = 0.085, respectively), whereas f [HR 0.825, (95% CI, 0.720 to 0.947), *p* = 0.006] remained a predictor of the disease.

**Table 3:** Cox regression analysis of the associations between DWI parameters and ESKD.

	Model 1		Model 2		Model 3	
	HR (95% CI)	*p*	HR (95% CI)	*p*	HR (95% CI)	*p*
ADC_T_	0.947(0.918 to 0.977)	0.001	0.961(0.926 to 0.998)	0.039	0.965(0.927 to 1.003)	0.072
D	0.92(0.884 to 0.958)	<0.001	0.95 (0.903 to 1.000)	0.049	0.955(0.906 to 1.006)	0.085
D*	0.951(0.895 to 1.012)	0.112	1.006(0.932 to 1.086)	0.875	1.015(0.939 to 1.097)	0.706
f	0.844(0.769 to 0.927)	<0.001	0.835(0.743 to 0.938)	0.002	0.825(0.720 to 0.947)	0.006

Model 1 was unadjusted; Model 2 was adjusted for age, sex, and baseline eGFR; Model 3 was adjusted for age, sex, baseline eGFR and albuminuria; eGFR, estimated glomerular filtration rate; ADC_T_, total apparent diffusion coefficient; D, true diffusion coefficient; D*, pseudo-diffusion coefficient; f, perfusion fraction.

## Discussion

There is growing interest in determining whether noninvasive imaging techniques can identify kidney injury, predict kidney function and accelerate the translation of multiparametric MRI of the kidneys into clinical practice (15). In our cohort of 62 patients followed up for 5 years, our study demonstrated that IVIM-DWI is capable of detecting early fibrosis and monitoring kidney prognosis in CKD patients. ADC_T_ and D were found to be noninvasive parameters that can be used to identify mild fibrosis in CKD patients. More surprisingly, the combination of baseline eGFR and MRI biomarkers seemed to predict pathological factors in the diagnosis of kidney dysfunction. Furthermore, f may be a stable parameter for independently predicting the progression of CKD after adjustment for baseline variables.

Although renal fibrosis is a key driver of the progression of CKD, the implementation of renal biopsy for assessing this condition is limited by its invasiveness, sampling bias and unsuitability for performing longitudinal follow-up. Our group demonstrated that both the ADC_T_ and f were positively correlated with the peritubular capillary density. On this basis, we present a 3D parametric picture of the peritubular capillary density with ADC_T_/f and the eGFR (9). Therefore, we further investigated the value of IVIM-DWI in identifying patients at high risk of chronic kidney disease progression. The eGFR and degree of albuminuria have been shown to be capable of predicting CKD prognosis; nevertheless, the ability of clinicians to predict events in those with established CKD within any given set of eGFR and urine albumin–creatinine ratio (UACR) categories has not been formally studied (16). To our knowledge, no similar studies have compared MRI biomarkers and pathology in the progression of CKD. Our study aimed to explore the value of imaging versus clinical and pathological factors in predicting ESKD in CKD patients to address the gap between clinical, pathological and imaging findings.

Diffusion MRI is widely used to assess kidney microstructure and microcirculation by measuring water movement. In IVIM-DWI, multiple b values are fit into a biexponential decay equation to separate perfusion from diffusion, yielding four characteristic variables: ADC_T_, D, D* and f. Although both ADC_T_ and D reflect the diffusion of water molecules, the ADC_T_ is thought to be affected by perfusion (17), D* is proportional to the mean capillary segment length and blood velocity, and f reflects the capillary density within the tissue since it is correlated with the general blood flow of the kidney (18, 19). In agreement with existing studies, including animal and human studies, our research revealed that IVIM-DWI parameters were negatively correlated with the area of renal fibrosis (19–21). Furthermore, we demonstrated that the performance of the ADC_T_ and D in discriminating ≤25 and >25% interstitial extracellular matrix volume was good, whereas the discriminatory effects of D* and f were not statistically significant. Zhu et al. (22) reported that there were no significant correlations between *f* values and histology, which is similar to our results; however, the results from existing studies are controversial (5, 23). This may be explained by the fact that changes in water molecules are more sensitive to pathophysiological changes in the kidneys in early fibrosis than changes in the microcirculation of the blood. Mao et al. (24) and Liang et al. (25) reported that IVIM-DWI is sensitive for detecting underlying pathologic injury in early CKD patients. Feng et al. (26) and Deng et al. (27) reported that IVIM-DWI parameters may serve as indicators for detecting early-stage kidney changes in diabetic patients. Given the limited population with >50% areas of fibrosis, we did not assess severe fibrosis further in our study.

The results of existing longitudinal follow-up studies on whether IVIM-DWI can predict renal function outcomes are controversial. Berchtold et al. (28) reported that the cortico-medullary difference in ADC_T_ is a predictor of renal function decline in a mixed population of 197 CKD and kidney allograft patients followed up for 5 years. In contrast, Sugiyama et al. (29) reported that reduced oxygenation but not fibrosis, as defined by functional MRI, that is, the blood-oxygen-level-dependent (BOLD) signal but not the ADC_T_, could predict long-term progression in a cohort of 91 CKD patients. In our study, compared with other parameters, the ADC_T_ showed similar performance in predicting ESKD, which may be explained by the fact that the ADC_T_ is affected by diffusion and perfusion simultaneously. The measurement of the D* value mostly depends on the use of lower b values (≤200 s/mm^2^), which may be easily influenced by noise and therefore result in lower stability and repeatability for this value (30). However, a previous study reported that the multiplication of f and D* can produce high-resolution brain perfusion maps in various brain diseases, which could eliminate some correlated uncertainties potentially present in both f and D* (31, 32). Joo et al. (33) also confirmed that fD* had good stability, allowing early prediction of the tumor response to vascular disrupting agent treatment. This is a new way to verify the value of D* in the prognosis of CKD patients.

As fibrosis worsens, there is progressive extracellular matrix deposition and capillary loss; unsurprisingly, the corresponding representative factors D and f play important roles in kidney disease prognosis. The novel finding in our study was that the combination of baseline eGFR and MRI biomarkers predicted kidney outcomes in CKD patients in a manner similar to or even better than the use of pathology parameters alone. On this basis, the next step was to determine whether follow-up MRI data could be used to assess kidney disease prognosis over at least the next 5 years. Zhang et al. (34) reported that IVIM-derived D and DTI-derived FA values were better than other parameters for evaluating early kidney impairment in patients with diabetes, which showed that D has good stability in detecting early renal injury. Berchtold et al. (35) demonstrated that a change in the ADC_T_ was more sensitive in detecting greater areas of kidney fibrosis in transplant patients than a change in the eGFR was in a longitudinal study of 19 kidney transplant recipients who had undergone serial kidney biopsies and MRI scans an average of 1.7 years apart. The value of IVIM-DWI needs to be explored in depth not only for determining the prognosis of kidney disease but also in combination with clinical trials on interventional drugs.

A greater degree of albuminuria is a risk factor for a reduced GFR and progression to ESKD (36). Additionally, significant correlations between the mean renal ADC_T_ value and excreted urine albumin have been demonstrated (6). However, importantly, our study indicated that albuminuria was not a good, independent predictor of the progression of CKD and even acted as a confounding factor for the association between MRI biomarkers and kidney dysfunction. This finding is consistent with that of an exploratory study by Srivastava et al. (37). We attribute this result to the heterogeneity of CKD, by which the degree of albuminuria in the participants could vary widely.

There are several limitations of our study. First, the accuracy of the predictors identified in this study was not externally validated because of the small sample size of a single center. Further multicenter studies with large sample sizes should be conducted to verify our results. Second, we ignored the effect of drugs on the eGFR during follow-up. The study population could not be characterized by a single disease, which may have resulted in different kidney outcomes due to the different medication regimens used. Third, the ROIs were delineated manually, resulting in a relatively subjective assessment that is infeasible for large-scale applicability in clinical practice.

In conclusion, our study provides histological evidence that IVIM-DWI can be used to noninvasively monitor kidney prognosis during long-term follow-up in CKD patients. These findings have important clinical value for the dynamic follow-up assessment of kidney disease prognosis.

## Data Availability

The datasets generated or analyzed during the study are available from the corresponding author upon reasonable request.

## References

[1] Chen TK Knicely DH Grams ME. Chronic kidney disease diagnosis and management: a review. JAMA. (2019) 322:1294–304. doi: 10.1001/jama.2019.14745, PMID: 31573641 PMC7015670

[2] Astor BC Matsushita K Gansevoort RT van der Velde M Woodward M Levey AS. Lower estimated glomerular filtration rate and higher albuminuria are associated with mortality and end-stage renal disease. A collaborative meta-analysis of kidney disease population cohorts. Kidney Int. (2011) 79:1331–40. doi: 10.1038/ki.2010.550, PMID: 21289598 PMC3917543

[3] Gansevoort RT Matsushita K van der Velde M Astor BC Woodward M Levey AS. Lower estimated GFR and higher albuminuria are associated with adverse kidney outcomes. A collaborative meta-analysis of general and high-risk population cohorts. Kidney Int. (2011) 80:93–104. doi: 10.1038/ki.2010.531, PMID: 21289597 PMC3959732

[4] van der Velde M Matsushita K Coresh J Astor BC Woodward M Levey A. Lower estimated glomerular filtration rate and higher albuminuria are associated with all-cause and cardiovascular mortality. A collaborative meta-analysis of high-risk population cohorts. Kidney Int. (2011) 79:1341–52. doi: 10.1038/ki.2010.536, PMID: 21307840

[5] Mao W Zhou J Zeng M Ding Y Qu L Chen C. Chronic kidney disease: pathological and functional evaluation with intravoxel incoherent motion diffusion-weighted imaging. J Magn Reson Imaging. (2018) 47:1251–9. doi: 10.1002/jmri.25861, PMID: 28940646

[6] Caroli A Schneider M Friedli I Ljimani A De Seigneux S Boor P. Diffusion-weighted magnetic resonance imaging to assess diffuse renal pathology: a systematic review and statement paper. Nephrol Dial Transplant. (2018) 33:ii29–40. doi: 10.1093/ndt/gfy163, PMID: 30137580 PMC6106641

[7] van Baalen S Leemans A Dik P Lilien MR Ten Haken B Froeling M. Intravoxel incoherent motion modeling in the kidneys: comparison of mono-, bi-, and triexponential fit. J Magn Reson Imaging. (2017) 46:228–39. doi: 10.1002/jmri.25519, PMID: 27787931 PMC5484284

[8] Zhang J Zhang LJ. Functional MRI as a tool for evaluating interstitial fibrosis and prognosis in kidney disease. Kidney Dis (Basel). (2020) 6:7–12. doi: 10.1159/000504708, PMID: 32021869 PMC6995954

[9] Zhang J Yu Y Liu X Tang X Xu F Zhang M. Evaluation of renal fibrosis by mapping histology and magnetic resonance imaging. Kidney Dis (Basel). (2021) 7:131–42. doi: 10.1159/000513332, PMID: 33824869 PMC8010230

[10] Eisenberger U Binser T Thoeny HC Boesch C Frey FJ Vermathen P. Living renal allograft transplantation: diffusion-weighted MR imaging in longitudinal follow-up of the donated and the remaining kidney. Radiology. (2014) 270:800–8. doi: 10.1148/radiol.13122588, PMID: 24475796

[11] Sułkowska K Palczewski P Furmańczyk-Zawiska A Perkowska-Ptasińska A Wójcik D Szeszkowski W. Diffusion weighted magnetic resonance imaging in the assessment of renal function and parenchymal changes in chronic kidney disease: a preliminary study. Ann Transplant. (2020) 25:e920232. doi: 10.12659/AOT.920232, PMID: 32123153 PMC7069451

[12] Wang Y Zheng C Xu F Liu Z. Urinary fibrinogen and renal tubulointerstitial fibrinogen deposition: discriminating between primary FSGS and minimal change disease. Biochem Biophys Res Commun. (2016) 478:1147–52. doi: 10.1016/j.bbrc.2016.08.083, PMID: 27539323

[13] Wang W Yu Y Wen J Zhang M Chen J Cheng D. Combination of functional magnetic resonance imaging and histopathologic analysis to evaluate interstitial fibrosis in kidney allografts. Clin J Am Soc Nephrol. (2019) 14:1372–80. doi: 10.2215/CJN.00020119, PMID: 31416890 PMC6730521

[14] Working Group of the International IgA Nephropathy Network and the Renal Pathology Society Cattran DC Coppo R Cook HT Feehally J Roberts IS. The Oxford classification of IgA nephropathy: rationale, clinicopathological correlations, and classification. Kidney Int. (2009) 76:534–45. doi: 10.1038/ki.2009.243, PMID: 19571791

[15] Francis ST Selby NM Taal MW. Magnetic resonance imaging to evaluate kidney structure, function, and pathology: moving toward clinical application. Am J Kidney Dis. (2023) 82:491–504. doi: 10.1053/j.ajkd.2023.02.007, PMID: 37187282

[16] Kadatz MJ Lee ES Levin A. Predicting progression in CKD: perspectives and precautions. Am J Kidney Dis. (2016) 67:779–86. doi: 10.1053/j.ajkd.2015.11.007, PMID: 26725311

[17] Leung G Kirpalani A Szeto SG Deeb M Foltz W Simmons CA. Could MRI be used to image kidney fibrosis? A review of recent advances and remaining barriers. Clin J Am Soc Nephrol. (2017) 12:1019–28. doi: 10.2215/CJN.07900716, PMID: 28298435 PMC5460707

[18] Liang L Chen WB Chan KW Li YG Zhang B Liang CH. Using intravoxel incoherent motion MR imaging to study the renal pathophysiological process of contrast-induced acute kidney injury in rats: comparison with conventional DWI and arterial spin labelling. Eur Radiol. (2016) 26:1597–605. doi: 10.1007/s00330-015-3990-y, PMID: 26373761

[19] Cai XR Yu J Zhou QC Du B Feng YZ Liu XL. Use of intravoxel incoherent motion MRI to assess renal fibrosis in a rat model of unilateral ureteral obstruction. J Magn Reson Imaging. (2016) 44:698–706. doi: 10.1002/jmri.25172, PMID: 26841951

[20] Hueper K Khalifa AA Bräsen JH Vo Chieu VD Gutberlet M Wintterle S. Diffusion-weighted imaging and diffusion tensor imaging detect delayed graft function and correlate with allograft fibrosis in patients early after kidney transplantation. J Magn Reson Imaging. (2016) 44:112–21. doi: 10.1002/jmri.25158, PMID: 26778459

[21] Friedli I Crowe LA Berchtold L Moll S Hadaya K de Perrot T. New magnetic resonance imaging index for renal fibrosis assessment: a comparison between diffusion-weighted imaging and T1 mapping with histological validation. Sci Rep. (2016) 6:30088. doi: 10.1038/srep30088, PMID: 27439482 PMC4954968

[22] Zhu J Chen A Gao J Zou M Du J Wu PY. Diffusion-weighted, intravoxel incoherent motion, and diffusion kurtosis tensor MR imaging in chronic kidney diseases: correlations with histology. Magn Reson Imaging. (2024) 106:1–7. doi: 10.1016/j.mri.2023.07.002, PMID: 37414367

[23] Mao W Zhou J Zeng M Ding Y Qu L Chen C. Intravoxel incoherent motion diffusion-weighted imaging for the assessment of renal fibrosis of chronic kidney disease: a preliminary study. Magn Reson Imaging. (2018) 47:118–24. doi: 10.1016/j.mri.2017.12.010, PMID: 29217491

[24] Mao W Ding Y Ding X Fu C Cao B Kuehn B. Capability of arterial spin labeling and intravoxel incoherent motion diffusion-weighted imaging to detect early kidney injury in chronic kidney disease. Eur Radiol. (2023) 33:3286–94. doi: 10.1007/s00330-022-09331-z, PMID: 36512040

[25] Liang P Yuan G Li S He K Peng Y Hu D. Non-invasive evaluation of the pathological and functional characteristics of chronic kidney disease by diffusion kurtosis imaging and intravoxel incoherent motion imaging: comparison with conventional DWI. Br J Radiol. (2023) 96:20220644. doi: 10.1259/bjr.20220644, PMID: 36400040 PMC10997028

[26] Feng YZ Chen XQ Yu J Liu XL Cheng ZY Ren WW. Intravoxel incoherent motion (IVIM) at 3.0 T: evaluation of early renal function changes in type 2 diabetic patients. Abdom Radiol (NY). (2018) 43:2764–73. doi: 10.1007/s00261-018-1555-7, PMID: 29525883

[27] Deng Y Yang B Peng Y Liu Z Luo J Du G. Use of intravoxel incoherent motion diffusion-weighted imaging to detect early changes in diabetic kidneys. Abdom Radiol (NY). (2018) 43:2728–33. doi: 10.1007/s00261-018-1521-4, PMID: 29541833

[28] Berchtold L Crowe LA Combescure C Kassaï M Aslam I Legouis D. Diffusion-magnetic resonance imaging predicts decline of kidney function in chronic kidney disease and in patients with a kidney allograft. Kidney Int. (2022) 101:804–13. doi: 10.1016/j.kint.2021.12.014, PMID: 35031327

[29] Sugiyama K Inoue T Kozawa E Ishikawa M Shimada A Kobayashi N. Reduced oxygenation but not fibrosis defined by functional magnetic resonance imaging predicts the long-term progression of chronic kidney disease. Nephrol Dial Transplant. (2020) 35:964–70. doi: 10.1093/ndt/gfy324, PMID: 30418615

[30] Cohen AD Schieke MC Hohenwalter MD Schmainda KM. The effect of low b-values on the intravoxel incoherent motion derived pseudodiffusion parameter in liver. Magn Reson Med. (2015) 73:306–11. doi: 10.1002/mrm.25109, PMID: 24478175 PMC4317387

[31] Federau C O'Brien K Meuli R Hagmann P Maeder P. Measuring brain perfusion with intravoxel incoherent motion (IVIM): initial clinical experience. J Magn Reson Imaging. (2014) 39:624–32. doi: 10.1002/jmri.24195, PMID: 24068649

[32] Le Bihan D Turner R. The capillary network: a link between IVIM and classical perfusion. Magn Reson Med. (1992) 27:171–8. doi: 10.1002/mrm.1910270116, PMID: 1435202

[33] Joo I Lee JM Han JK Choi BI. Intravoxel incoherent motion diffusion-weighted MR imaging for monitoring the therapeutic efficacy of the vascular disrupting agent CKD-516 in rabbit VX2 liver tumors. Radiology. (2014) 272:417–26. doi: 10.1148/radiol.14131165, PMID: 24697148

[34] Zhang H Wang P Shi D Yao X Li Y Liu X. Capability of intravoxel incoherent motion and diffusion tensor imaging to detect early kidney injury in type 2 diabetes. Eur Radiol. (2022) 32:2988–97. doi: 10.1007/s00330-021-08415-6, PMID: 35031840

[35] Berchtold L Crowe LA Friedli I Legouis D Moll S de Perrot T. Diffusion magnetic resonance imaging detects an increase in interstitial fibrosis earlier than the decline of renal function. Nephrol Dial Transplant. (2020) 35:1274–6. doi: 10.1093/ndt/gfaa007, PMID: 32160279

[36] Kidney Disease: Improving Global Outcomes (KDIGO) Glomerular Diseases Work Group. KDIGO. Clinical practice guideline for the Management of Glomerular Diseases. Kidney Int. (2021) 100:S1–S276. doi: 10.1016/j.kint.2021.05.021 34556256

[37] Srivastava A Cai X Lee J Li W Larive B Kendrick C. Kidney functional magnetic resonance imaging and change in eGFR in individuals with CKD. Clin J Am Soc Nephrol. (2020) 15:776–83. doi: 10.2215/CJN.13201019, PMID: 32345747 PMC7274274

