# Supplementary material for: Utility of the combination of IVIM-DWI MRI and baseline eGFR for identifying a high risk of chronic kidney disease progression
Source: Front Med (Lausanne). 2025 Feb 19;12:1532210. doi: 10.3389/fmed.2025.1532210 (PMC11880002; doi:10.3389/fmed.2025.1532210)

**Supplemental Table 1 Parameters for IVIM-DWI MRI**

**Supplemental Table 2 Correlation of IVIM-DWI parameters with proteinuria, baseline eGFR, eGFR slope and interstitial extracellular matrix volume**

**Supplemental Table 3 The ROC analysis of IVIM-DWI parameters to diagnose early fibrosis in CKD patients**

**Supplemental Figure 1 Flow chart of study**

**Supplemental Figure 2 Different causes of CKD in the study**

**Supplemental Table 1 Parameters for IVIM-DWI MRI**

| Parameters for IVIM-DWI MRI |  |
| --- | --- |
| Voxel size (mm^3^) | 1.7×2.4×6 |
| Echo time (ms) | 87 |
| Repetition time (ms) | 3500 |
| Slice thickness (mm) | 6 |
| Intersection gap (mm) | 1 |
| matrix | 224×128 |
| Flip angle | 90 |
| FOV (cm²) | 38.0×30.4 |
| b-values (s/mm²) | 10,30,50,70,100,150,200,400, 800,1000 |
| NEX (Number of excitations) | 2 |

**Supplemental Table 2 Correlation of IVIM-DWI parameters with proteinuria, baseline eGFR, eGFR slope and interstitial extracellular matrix volume**

|  | Proteinuria | | Baseline eGFR | | eGFR slope | | Interstitial extracellular matrix volume | |
| --- | --- | --- | --- | --- | --- | --- | --- | --- |
|  | r_s_ | p | r_s_ | p | r_s_ | p | r_s_ | p |
| ADC_T_ | -0.12 | 1 | 0.42 | 0.028 | 0.32 | 0.308 | -0.43 | 0.028 |
| D | -0.07 | 1 | 0.58 | 0.028 | 0.38 | 0.056 | -0.46 | 0.028 |
| D* | 0.10 | 1 | 0.44 | 0.028 | 0.22 | 1 | -0.32 | 0.308 |
| f | -0.28 | 0.812 | 0.17 | 1 | 0.54 | 0.028 | -0.25 | 1 |

**Supplemental Table 3 The ROC analysis of IVIM-DWI parameters to diagnose early fibrosis in CKD patients**

| Measures | Cutoff | AUC (95%CI) | Sensitivity | Specificity | P |
| --- | --- | --- | --- | --- | --- |
| ADC_T_ | 1.85 | 0.715 (0.577 to 0.853) | 87.5% | 54.5% | 0.005 |
| D | 1.46 | 0.72 (0.585 to 0.855) | 60% | 81.8% | 0.004 |
| D* | / | / | / | / | 0.181 |
| Fp | / | / | / | / | 0.104 |

**Supplemental Figure 1 Flow chart of study**

**
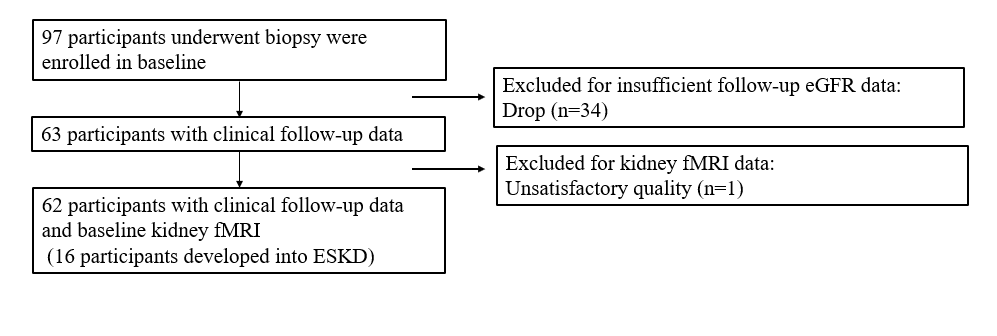
**

**Supplemental Figure 2 Different causes of CKD in the study**


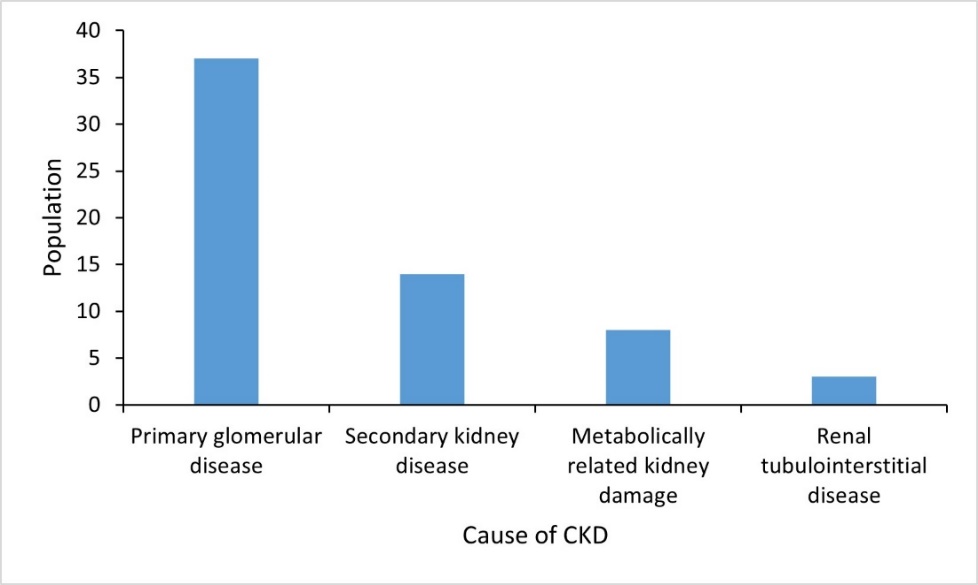

Supplement: Supplementary file 1 [file Data_Sheet_1.docx]
